# Asiatic Cholera: Its Origin and Spread in Asia, Africa, and Europe; Introduction into America through Canada; Remote and Proximate Causes, Symptoms, and Pathology, and the Various Modes of Treatment Analysed

**Published:** 1866-08

**Authors:** 


Asiatic Cholera: Its Origin and Spread in Asia, Africa, and Europe; Introduction into America through Canada; Remote and Proximate Causes, Symptoms, and Pathology, and the Various Modes of Treatment Analyzed. By R. Nelson, M.D., Health Commissioner during the first two invasions, 1832-4; President of the Medical Board for the District of Montreal. W. A. Townsend, Publisher, New York. 1866.

This is a duodecimo volume of 206 pages. Its copious title page sufficiently indicates its contents. Those who wish to accumulate all the current literature on the subject of cholera will procure the present work. But we cannot approve of the author's views, either in relation to the pathology or treatment of the disease of which he treats.

